# Open-label feasibility study of pazopanib, carboplatin, and paclitaxel in women with newly diagnosed, untreated, gynaecologic tumours: a phase I/II trial of the AGO study group

**DOI:** 10.1038/bjc.2011.608

**Published:** 2012-01-12

**Authors:** A du Bois, I Vergote, P Wimberger, I Ray-Coquard, P Harter, L B Curtis, I Mitrica

**Affiliations:** 1 Departments of Gynecology and Gynecologic Oncology, Kliniken Essen Mitte, Henricistrasse 92, 45136 Essen, Germany; 2 Department of Gynecologic Oncology, University Hospitals Leuven, Leuven, Belgium; 3 Department of Gynecology and Obstetrics, University of Duisburg-Essen, Essen, Germany; 4 Centre Leon Bérard, Lyon, France; 5 GlaxoSmithKline, Brentford, London, UK; 6 GlaxoSmithKline, Research Triangle Park, NC, USA

**Keywords:** ovarian cancer, pazopanib, phase I study

## Abstract

**Introduction:**

Although most patients with advanced gynaecologic malignancies respond to first-line treatment with platinum-taxane doublets, a significant proportion of patients relapse. Combining targeted agents that have non-overlapping mechanisms of action with chemotherapy may potentially increase the disease-free interval. Accordingly, this study evaluated the feasibility of combining pazopanib, an oral angiogenesis inhibitor, with paclitaxel and carboplatin.

**Methods:**

This open-label, phase I/II study planned to evaluate the safety and efficacy of paclitaxel 175 mg m^–2^ plus carboplatin (AUC5 (Arm A) or AUC6 (Arm B)) once in every 3 weeks for up to six cycles with either 800 or 400 mg per day pazopanib.

**Results:**

Dose-limiting toxicities (DLTs) were observed in two of the first six patients enrolled at pazopanib 800 mg plus paclitaxel 175 mg m^–2^ plus carboplatin AUC5. Of the six patients enrolled in the next and lowest dosing level planned in the study, pazopanib 400 mg plus paclitaxel 175 mg m^–2^ plus carboplatin AUC5, two patients also experienced DLTs and the study was terminated. Two of the 4 DLTs observed overall were gastrointestinal perforations. Severe myelotoxicity was reported in 6 of 12 patients.

**Conclusion:**

Combining either 800 or 400 mg per day pazopanib with standard carboplatin/paclitaxel chemotherapy is not a feasible treatment option.

Platinum-taxane doublets are widely used as a standard first-line treatment for patients with advanced gynaecologic malignancies (du Bois *et al*, 2003; Greer *et al*, 2008; Morgan *et al*, 2008). However, a high proportion of patients eventually relapse. One of the clinical approaches to increase the duration of disease control has been to identify new agents with a non-overlapping mechanism of action and demonstrated single-agent antitumor activity to combine with platinum and paclitaxel.

Translational data suggest that angiogenesis has a critical role in the growth of ovarian tumours and is therefore a potentially viable therapeutic target (Yamamoto *et al*, 1997; Cooper *et al*, 2002). Thus, combining an active antiangiogenic agent with standard chemotherapy may potentially improve tumour control and provide sustained benefit. Indeed, this approach has been validated in controlled phase III trials in patients with advanced solid tumours (Sandler *et al*, 2006; Reck *et al*, 2009). More recently, two phase III trials reported that adding bevacizumab to standard chemotherapy in women with newly diagnosed ovarian cancer significantly improved progression-free survival (Burger *et al*, 2010; Kristensen *et al*, 2011) and overall survival for a subgroup of patients with residual disease after initial surgery (Kristensen *et al*, 2011).

Pazopanib is an oral angiogenesis inhibitor targeting vascular endothelial growth factor receptors, platelet-derived growth factor receptors, and c-Kit (Sonpavde and Hutson, 2007) with demonstrated single-agent activity in renal cell carcinoma (Friedlander *et al*, 2010) and soft tissue sarcoma (Van Der Graaf *et al*, 2011). In addition, preliminary evidence of clinical activity associated with pazopanib has been observed in breast cancer (Taylor *et al*, 2010), thyroid tumours (Iwamoto *et al*, 2010), and gynaecologic tumours including recurrent ovarian disease (Friedlander *et al*, 2010) and cervical cancer (Monk *et al*, 2010). A maximum tolerated regimen for this combination had previously been identified in patients with solid tumours and up to three previous treatments as pazopanib 200 mg daily with paclitaxel 175 mg m^–2^ and carboplatin at area under the curve 5 (AUC5) given every 3 weeks; however, the optimal dosing regimen was not established in this setting of untreated gynaecologic cancers using a short-term chemotherapy regimen.

Accordingly, this phase I/II study (VEG110190; clinicaltrial.gov identifier NCT00561795) explored the feasibility of combining pazopanib with the standard regimen of paclitaxel and carboplatin as first-line treatment in patients with advanced gynaecologic tumours.

## Patients and Methods

### Patients

This study enrolled adult women (⩾18 years of age) with newly diagnosed, measurable or non-measurable advanced gynaecologic tumours, for whom carboplatin-paclitaxel chemotherapy was indicated. Additional eligibility criteria included a performance status of 0 or 1 on the Eastern Cooperative Oncology Group (ECOG) scale and adequate major system/organ function.

### Study design, treatment, and assessment

This open-label, phase I/II study explored the safety and tolerability of adding pazopanib to a standard combination of paclitaxel and carboplatin in patients with previously untreated, advanced gynaecologic tumours. It was planned that a minimum of 12 and a maximum of 46 women would be enrolled. The study planned to test two treatment arms: patients enrolled in arm A received paclitaxel 175 mg m^–2^ and carboplatin AUC5 every 3 weeks for up to 6 cycles plus daily pazopanib; if arm A was successful, patients enrolled in arm B would receive paclitaxel 175 mg m^–2^ and carboplatin AUC6 every 3 weeks for up to 6 cycles plus daily pazopanib. Within each arm, two dosing levels of pazopanib (800 and 400 mg per day) were planned to be tested. Pazopanib dosing was started at 800 mg per day, and if not adequately tolerated, could be reduced to 400 mg per day for individual patients, or if necessary, reduced in the subsequent arm. Tolerability was assessed in accordance with standard, predefined clinical criteria for dose-limiting toxicity. Adverse events were graded according to Common Terminology Criteria for Adverse Events (version 3.0) and coded to the preferred term level using the Medical Dictionary for Regulatory Activities.

### Study objectives

The primary objectives of the study were to develop a feasible dose and schedule, and to assess the safety and tolerability of combining pazopanib with standard carboplatin/paclitaxel chemotherapy in women with previously untreated, advanced gynaecologic cancers. Secondary objectives included evaluation of tumour response rate in patients with measurable disease, 18-week progression-free survival, and cancer antigen (CA-125) response rate in patients with elevated levels at baseline.

## Results

This study enrolled 12 Caucasian women with a mean age of 54 years (range 39–65 years) and ECOG performance status of 0 (eight patients; 67%) or 1 (four patients; 33%). The safety population included all 12 patients enrolled in arm A, all of whom received at least one dose of study medication.

### Feasibility/tolerability

Of the six patients enrolled in the pazopanib 800 mg (once daily) plus paclitaxel 175 mg m^–2^ and carboplatin AUC5 (q 3 weeks × six cycles for both) cohort, two patients experienced DLTs. These included grade 5 ileal perforation (one patient), which eventually led to death during the study, and grade 3 abdominal cramps (one patient), which led to dose reduction. The patient with ileal perforation had extensive tumour involvement of the small bowel mesentery, and a residual tumour >2 cm in size after surgery. Accordingly, the dose of pazopanib was reduced to 400 mg in the six additional patients enrolled and treated with paclitaxel 175 mg m^–2^ and carboplatin AUC5. However, two patients in this cohort also experienced DLTs, which included grade 4 intestinal perforation (one patient) and grade 2 skin necrosis (one patient), both leading to discontinuation of treatment. Per protocol, pazopanib was not further dose reduced, and arm B, which was to explore a combination regimen with a higher dose of carboplatin (AUC6), was not evaluated. The study was closed because of excessive toxicity, and a maximum tolerated regimen was not identified.

### Safety

Overall, 10 of the 12 patients enrolled in this study discontinued treatment. Seven patients (58%) discontinued because of treatment-related adverse events, and treatment for three patients was discontinued prematurely when the study closed. Myelotoxicity was the most common AE (Table 1) and the leading cause of treatment discontinuation. Overall, eight patients (67%) experienced serious treatment-related adverse events, which included neutropenia in six patients (50%) and gastrointestinal perforations in two patients (17%).

### Efficacy

Efficacy was not evaluated because of early treatment discontinuation in most patients and small patient numbers. No patients progressed on receiving study treatment.

## Discussion

Pazopanib 800 mg once daily or 400 mg once daily administered concurrently with standard paclitaxel and carboplatin chemotherapy is not a feasible regimen in patients with newly diagnosed gynaecologic malignancies, because of unacceptable toxicity. A pazopanib dose of 200 mg once daily was not considered clinically meaningful because drug exposure would be subtherapeutic in many patients, and was therefore not further explored (Hurwitz *et al*, 2009). The high frequency and severity of toxicities reported in this study may be related to drug interactions between pazopanib and paclitaxel and/or carboplatin that result in undesirably high levels of chemotherapy agents in patients. Indeed, a recent phase I study (VEG105427, Part 2) that evaluated this combination in patients with advanced solid tumours showed that pazopanib decreased the clearance of paclitaxel, increased the AUC of carboplatin, and increased maximum concentration (*C*_max_) of both paclitaxel and carboplatin (Burris *et al*, 2009). Similar toxicity data have been reported in studies attempting to combine other antiangiogenic agents with a platinum-taxane doublet. For example, a phase II study of sorafenib in combination with paclitaxel 175 mg m^–2^ and carboplatin AUC5 was terminated because of excessive toxicities observed in the first four patients enrolled (Polcher *et al*, 2010). Likewise, a clinical trial exploring the addition of sunitinib to paclitaxel plus carboplatin chemotherapy concluded that this combination, although feasible, was difficult to administer (Heath *et al*, 2011). Had these safety issues and data on potential pharmacokinetic interaction been known at the time of design or conduct of the current study, a formal phase I dose-escalation study with a dense pharmacokinetic sampling schedule would have been considered as an alternative to the current phase I/II study.

Pazopanib and other antiangiogenic agents have demonstrated single-agent antitumor activity in multiple tumour types (Sleijfer *et al*, 2009; Altorki *et al*, 2010; Friedlander *et al*, 2010; Sternberg *et al*, 2010) and have shown preliminary evidence of antitumor activity in gynaecologic tumours (Friedlander *et al*, 2010). Although it may not be feasible to combine some of these agents with standard chemotherapy, the potential remains to increase the duration of disease-free survival by using these agents as a consolidation/maintenance monotherapy after tumour control has been achieved with standard treatment modalities. Indeed, in the recent report of the phase III GOG 0218 and ICON7 trials, the addition of adjuvant bevacizumab to chemotherapy plus bevacizumab consolidation significantly improved progression-free survival compared with chemotherapy alone or chemotherapy plus bevacizumab (Burger *et al*, 2010; Kristensen *et al*, 2011). Accordingly, an ongoing phase III, placebo-controlled trial led by Arbeitsgemeinschaft Gynäkologische Onkologie (AGO) is exploring the benefit of 24 months of pazopanib monotherapy in the maintenance setting in patients with stage II–IV ovarian disease without disease progression after first-line chemotherapy (AGOOVAR16; clinicaltrials.gov identifier NCT00866697). This trial has already reached full recruitment with 900 patients, and results are awaited. In addition, combinations of other pazopanib regimens (e.g., intermittent) with other chemotherapy regimens (e.g., weekly carboplatin and/or paclitaxel) could still be feasible and are currently being tested in clinical studies (Harter and Marth, 2011; National Cancer Institute, 2011).

## Figures and Tables

**Table 1:** Summary of adverse events reported in ⩾2 patients in any arm

	**Paclitaxel 175 mg m^–2^ plus carboplatin AUC5**	
**AEs regardless of causality, *n* (%)**	**Pazopanib 800 mg (*n*=6)**	**Pazopanib 400 mg (*n*=6)**
*Haematologic*		
Neutropenia	5 (83)	3 (50)
Thrombocytopenia	3 (50)	1 (17)
Haemoglobin decreased	1 (17)	2 (33)
		
*Nonhaematologic*		
Alopecia	4 (67)	3 (50)
Fatigue	4 (67)	4 (67)
Abdominal pain	3 (50)	2 (33)
Nausea	3 (50)	3 (50)
Nasopharyngitis	3 (50)	0
Diarrhoea	2 (33)	2 (33)
Vomiting	2 (33)	1 (17)

Abbreviations: AEs=adverse events; AUC=area under the curve.
